# Cognitive testing and the hazards of cut-offs

**DOI:** 10.1192/bja.2024.10

**Published:** 2025-01

**Authors:** Hugh Series, Alistair Burns

**Affiliations:** Consultant old age psychiatrist with Oxford Health NHS Foundation Trust and a member of the Law Faculty, University of Oxford, Oxford, UK. He has a particular interest in medical law, and the use of probability and statistics in legal cases. He regularly teaches law students as well as medical students and others. He is approved under section 12 of the Mental Health Act 1983 and is a member of the First-tier Tribunal (Mental Health) in England.; Emeritus Professor of Old Age Psychiatry in the Division of Neuroscience and Experimental Psychology, Faculty of Biology, Medicine and Health, University of Manchester, Manchester, UK, and an honorary consultant age psychiatrist with Oxford Health NHS Foundation Trust, Oxford, UK. Since 2010 he has acted as the National Clinical Director for Dementia (and Older People's Mental Health) for the NHS in England. He is approved under section 12 of the Mental Health Act 1983.

**Keywords:** Clinical outcomes measures, dementias/neurodegenerative diseases, epidemiology, statistical methodology, rating scales

## Abstract

The article reviews some basic statistical concepts used in medicine, including the mean, standard deviation, sensitivity and specificity. Using this background the authors describe how these can be applied to cognitive tests, taking the Montreal Cognitive Assessment (MoCA) as an example. Two different approaches to using the MoCA in diagnosing dementia are considered: one using a fixed cut-off score, the other taking account of normative data about the effects of age and educational level on MoCA scores. It is recommended that clinicians assessing cognitive function should not rely on a fixed cut-off score, but where possible compare the patient's result with those of people of comparable age and educational background, although normative data of this kind are not always available.

## LEARNING OBJECTIVES

After reading this article you will be able to:

- explain what makes a good cognitive test from a psychometric perspective
- explain why it matters to choose a test with high sensitivity and specificity
- explain why it matters to compare the patient's result with normative data, using the MoCA as an example.

In our previous article in this issue (Burns 2024) we discussed the role of neurocognitive testing in the assessment of fitness to stand trial, reviewing some of the shorter cognitive tests that are available and suggesting which to use and when. This second article considers the value – and hazards – of using cut-offs in such tests.

## Why use cognitive tests?

Cognitive assessments are used in a wide variety of clinical situations. In primary care they are helpful in identifying people who might benefit from referral to secondary care for further investigation, as well as being a helpful tool in reassuring the ‘worried well’ that their cognitive function may be no different from that of other people of their age. In accident and emergency departments they can help to identify delirium and dementia. In general medicine and neurology they can help to identify the development of dementia in conditions that are often regarded as primarily physical, such as multiple sclerosis or Parkinson's disease.

Making an assessment of cognitive function is an essential part of assessing and diagnosing dementia. In the context of a memory clinic, one is often trying to place a patient somewhere on a spectrum of cognitive impairment that runs from ‘within the normal range’ through ‘normal ageing’ (sometimes referred to as ‘age-associated cognitive impairment’ or ‘benign senescent forgetfulness’), to mild cognitive impairment, to dementia of one kind or another. There are a vast number of possible causes of cognitive impairment, both functional and organic. Making an assessment of the severity of the impairment is often critical to making an accurate diagnosis and offering appropriate advice and treatment.

Assessing cognitive function is key not only in diagnosing dementia, but also in tracking its progression. The degree of impairment is likely to affect the advice given and the medication offered. It may also assist in diagnosis: a cognitive impairment that shows no signs of progression over several years is less likely to be due to dementia than to some other cause. Some cognitive impairments following strokes may be very stable, and these are perhaps better described as ‘vascular cognitive impairment’ than as ‘vascular dementia’. Cognitive impairments may be seen in disorders that can improve, such as depression and psychotic disorders. If a cognitive impairment improves over time it is relatively unlikely to be due to dementia.

Many disciplines outside psychiatry make use of cognitive assessments. Neurologists, geriatricians and psychologists in particular are likely to employ them. In primary care, short cognitive assessments can be a helpful guide to referral.

## Cognitive tests as a diagnostic tool

### How they help

As we have seen, cognitive tests are a key part of a diagnostic assessment of dementia and in determining its severity. Given that impairment of cognitive function across multiple domains is a key element of the diagnosis of dementia it is difficult to see how a dementia diagnosis could be made without using some form of cognitive assessment.

### How they can hinder

Cognitive tests can be misleading. There can be substantial variations in the results on a given test from one occasion to another. Some of the variables arise within the patient (for example, change or fluctuation in disease severity, anxiety, tiredness, fear, inattention, side-effects of medication or alcohol, age), and some may be located in the test procedure (test–retest learning, approach of the examiner, test version used, scoring method, testing environment, distractions).

## The importance of normative data

In any measurement made in medicine, as doctors we need to know whether it is within the range expected, or is sufficiently abnormal to suggest that there may be a problem. So, we have to compare the person's result with those of a group of similar people. But similar in what way? That depends on what we think are the kinds of problems that might be present. For people with cognitive impairment, ‘similar’ would certainly include similar in age and education, both of which are known to have substantial effects on cognitive function. Many other variables might be relevant – cultural group, language spoken, physical illnesses, state of alertness, sensory impairments – but in practice it is not possible to take everything into account in a quantitative way, so we have to choose the best normative data available for the person's condition.

As doctors we consider a very wide range of test results in the course of our practice. In many cases, we rely on our knowledge of published normal values to determine whether a result is abnormal. This is particularly true in relation to blood tests, although even there, for some blood tests it is important to take into account age or racial group or time of day. There is sufficient consistency in many results between different groups of people that identifying an appropriate control group for a particular patient is often not necessary. The aim of this article is to explain why in relation to cognitive tests that approach is insufficient.

## Choosing a normative data-set

The perfect normative data-set would consist of a large number of people identical to the patient in every respect except the one under study. That is of course not possible in practice. We would suggest that age and educational level are key variables to consider, as both of those factors have substantial effects on cognitive performance.

For many other variables, one can try to minimise departure from norms by making adjustments to the test procedure. For example, if the person has poor hearing, ensure that they have their hearing aids (if used), that the room is quiet and that the assessor has the person's full attention. If the person has poor sight, ensure that they have their glasses, that the light is good and that they can see the test paper as necessary. If the person is anxious, consider whether having a trusted person with them would help. If the person has done the particular test before, consider whether there is an alternative version to use (many tests, including the MoCA, come in several versions to reduce test–retest learning effects).

## Comparing a test result with a normative score

### Above or below average?

The simplest, though not necessarily the best, way to use a test result is to compare it with the average score for a similar group of people.

This approach has the obvious shortcoming that in any population, half the population is above average and half below, although this should be qualified according to the type of average quoted. It is by definition true only of the median. Averages may be calculated as the mean, median or mode of a set of data.

The mean will be weighted by outlying values, which can pull the mean in either direction. This can be a particular problem in data-sets that are small or contain extreme outlying values. Some of those outliers may arise from errors in the data collection, and so researchers may reasonably choose to exclude some outliers. Nevertheless, the mean is used in many statistical analyses and is therefore an important and often-used figure.

The median is the middle value of a data-set (the 50th centile), and in the context of medical assessments may be a more useful measure as it is much less likely to be influenced by outliers.

The mode is the most common value, and less likely to be used in this type of analysis.

For many tests, there is not much good-quality normative data for different groups, so one has to use whatever is available.

### How far above or below? Standard deviation and variance

Simply deciding whether a result is above or below average is of limited help, unless one can say how far above or below it is. This can be measured by considering the spread of the data around the average. Two common measures of spread are standard deviation (s.d.) and variance (which is the square of the standard deviation). The standard deviation is the square root of the mean of the squared deviations of each value from the mean, sometimes referred to as the root mean square (r.m.s.) deviation. These numbers are key in many statistical tests. In considering them, it is essential to understand whether it is the variance or standard deviation that is quoted, as the size of the two numbers is likely to be very different.

Another statistic often used is the standard error, which is defined as the standard deviation of the sampling distribution. If the statistic is the sample mean, it is referred to as the standard error of the mean (s.e.m.). The sampling distribution of the mean is obtained by taking repeated samples from the same population and recording the mean of each sample. This forms a distribution with its own mean and variance. The variance of the sampling mean is equal to the variance of the original population divided by the sample size. For a given set of data, the s.e.m. is therefore likely to be much smaller than the s.d. (it will be the standard deviation of the sample divided by the square root of *n*, where *n* is the size of the sample). Studies may report results in terms of mean and either s.d. or s.e.m. (either as numbers in tables or as error bars on figures), so it is important to understand which is being used. Error bars showing s.e.m. will be much smaller than error bars showing s.d..

The s.d. is a particularly helpful statistic to consider in a population in which the variable is distributed normally or near normally (the bell-shaped function or ‘bell curve’). If the distribution of the variable is not normal, it may be possible to convert it to a near normal distribution, for example by using the log of the measurement.

**FIG 1:**
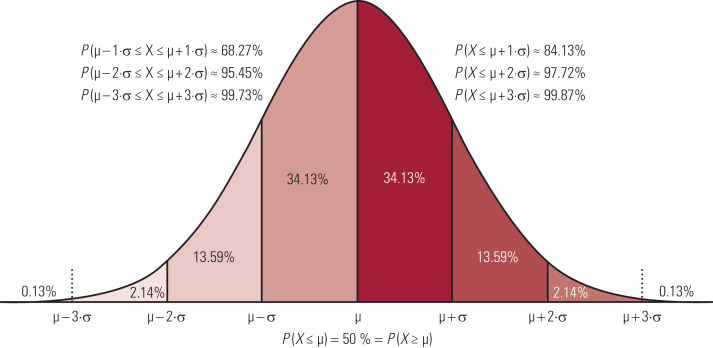
A graph of a normal distribution showing 1, 2 and 3 standard deviations (σ) from the mean (μ), where *P* is the probability of a value occurring at that deviation from the mean. Adapted from original by Wolfgang Kowarschick (https://commons.wikimedia.org/wiki/File:Normal_Distribution_Sigma.svg), under a Creative Commons licence (https://creativecommons.org/licenses/by-sa/4.0/deed.en).

Figure 1 shows an example of a normal distribution. Provided that the population from which the data are drawn is normal (an assumption that is often made about much biological data), it is possible to calculate how many per cent above or below the mean the test result lies. In many cases in medicine, a result is regarded as significant if it lies outside the central 95% of the population. There is a difference between a one-tailed calculation, in which one is interested only in deviations in one direction (the top or bottom 5% as the case may be), and two-tailed calculations, in which one is interested in deviations in either direction (values falling into either the top or the bottom 2.5%). It can be seen in Fig. 1 that 2.27% (0.13% + 2.14%) lie more than 2 s.d. below the mean; 68.27% lie within 1 s.d. either way of the mean, and 95.45% lie within 2 s.d. of the mean. A convenient figure to remember is that approximately 5% of data points lie more than 2 s.d. from the mean in a two-tailed test, and approximately 2.5% lie more than 2 s.d. below the mean in a one-tailed test.

A simple way to express data of this kind is in terms of centiles (or percentiles, which are the same thing). Imagine that there are 100 data points, one measurement from each of 100 individuals, lined up in order of magnitude. A person on the 50th centile is exactly in the middle (the median), whereas a person on the 5th centile is very low in the distribution, the fifth one along from the bottom value. The line marking 2 s.d. below the mean is at about the 2.5th centile.

## What is a ‘cut-off’?

A cut-off is a score level in a given test below which (or sometimes above which) it is suggested that an individual's score is significantly different from expected. If it is a diagnostic test, it is the level that divides those who are test positive (i.e. those who are identified by the test as having the disorder) from those who are test negative (i.e. those who are identified by the test as being free from the disorder).

But that simple idea conceals a number of questions:

- what is meant by ‘significant’?
- can any test be 100% reliable (or accurate)?
- what does reliable mean?
- since no test is entirely accurate, how do you take account of people you miss (false negatives) or those who are incorrectly diagnosed as having the condition (false positives)?

## How are cut-offs derived?

### Sensitivity and specificity

Consider a test that is being used to diagnose a specific condition. It is important to understand how accurate the test is. Accuracy is often considered in terms of the sensitivity and specificity of a test, although as we shall see some other calculations can be more useful.

Consider a test, T, used to diagnose a disorder, D. In a study examining the reliability of the test against some gold standard method of diagnosis in a total of A + B + C + D individuals, the results shown in Table 1 are obtained.

**TABLE 1:** In A + B + C + D individuals, the numbers with or without a disorder who test positive or negative in a diagnostic test

	With the disorder	Without the disorder	Row totals
Test positive	A (true positive)	B (false positive)	A + B
Test negative	C (false negative)	D (true negative)	C + D
Column totals	A + C	B + D	A + B + C + D

The sensitivity of the test is A/(A + C). In other words it is the proportion of all those who actually have the disease who test positive. It is the ability of the test to classify individuals correctly as ‘diseased’. It is the same as the true-positive rate. If a diagnostic test has a sensitivity (Sn) of 100%, then a negative (N) test result rules out (Out) the disorder (acronym ‘SnNOut’).

The specificity of the test is D/(B + D). In other words it is the proportion of all those who do not have the disease who test negative. It is the ability of the test to classify individuals correctly as disease-free. If a diagnostic test has a specificity (Sp) of 100%, then a positive (P) test result rules in (In) the disorder (acronym ‘SpPIn’).

Another way of considering the accuracy of a test is by using its positive and negative predictive value (PPV and NPV). The PPV is the probability that an individual with a positive test result truly has the condition. The NPV is the probability that an individual who tests negative truly does not have that condition. These are very useful probabilities to know if you are discussing a test result with the patient in a clinic, but they are less often quoted in validation studies than the sensitivity and specificity, and they are affected by the prevalence of the disease in the population studied:
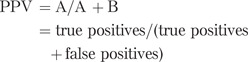

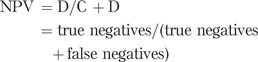


The term ‘false-positive rate’ is used in different ways, but often means 1 − specificity (= B/B + D), which is the meaning we have adopted here.

### ROC calculations

In practice, few clinicians have the time or access to the data to make these calculations in the course of a busy clinic. So it can be convenient to calculate a cut-off score for a test, which is a simple way of dividing those who are likely to have a condition from those who probably do not have it. A standard way of calculating a cut-off for a test is by means of a receiver operating characteristic (ROC) curve.

**FIG 2:**
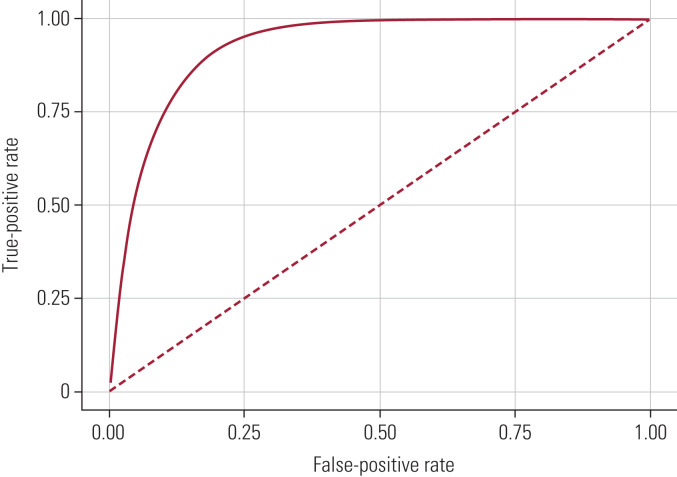
A receiver operating characteristic (ROC) curve of the true-positive rate (sensitivity) versus the false-positive rate (1 − specificity).

Figure 2 shows a ROC curve of the true-positive rate against the false-positive rate. As the cut-off score separating diseased from not diseased is adjusted, sensitivity and specificity are affected. With increasing sensitivity the true-positive rate improves, but the false-positive rate also increases. The broken line in the figure would indicate that the test is incapable of separating those with the disease from those without it. The further away the test (solid) line is from the broken line (or more accurately, the larger the area under the curve) the better the test is at separating those with the disorder from those without it. The optimal cut-off is the one that achieves the best compromise of sensitivity and specificity. That may depend on whether it is more important to detect as many cases as possible so that they can go on for further investigation (as in screening), or to be as confident as possible that anyone who tests positive actually has the disorder (which may have implications for treatment and prognosis). In the context of a memory clinic, it may be helpful to consider that expressing uncertainty about a diagnosis and waiting for some time to reassess the patient may be a better approach than to risk giving a patient what might turn out to be a wrong diagnosis of dementia, with all the very serious implications that that can have, including litigation and the patient's exposure to treatment that may in fact not be appropriate. For this reason, our preference is, if in doubt, to delay diagnosis and reassess after a period of time. This might be different if we had access to disease-modifying treatments, where a delay in diagnosis might affect long-term outcome.

Constructing and using ROC curves depends on some very complex considerations that are outside the scope of this article, but it is important to understand that the optimal balance of sensitivity and specificity will depend on the purpose for which the test is being used, and there is no single best solution. For screening tests, it is important to choose high sensitivity in order not to miss cases, but for prescribing treatments it is more important to choose high specificity in order to avoid giving potentially hazardous treatment to people who do not in fact have the disease.

### Significance

‘Significance’ is a widely used concept in probability theory that identifies how likely it is that a given result showing a difference from an expected value, such as the population mean, has arisen by chance. This has a clear application in a treatment study where one is interested in whether or not a new treatment is in fact effective, or whether in reality it is ineffective but by chance the trial happens to have shown a difference from the control group. But it also has application in diagnostic settings in which one wants to know how likely it is that a given test result is significantly different from normal.

By convention, a 5% level of significance has come to be accepted as a convenient indication that it is unlikely that a particular result has arisen by chance and is therefore more likely to reflect a true (‘significant’) difference. The 5% level was described by Sir Ronald Fisher in 1925 in his influential book *Statistical Methods for Research Workers* (Fisher 1925). Although the 5% figure is a useful and widely accepted marker, it is an arbitrary one. As noted above, there is a difference between a 5% level applied in a two-tailed test from a 5% level applied in a one-tailed test. In a memory clinic, one is concerned with cognitive test results that are below expected values, and so a one-tailed test is appropriate.

## The Montreal Cognitive Assessment (MoCA)

As an example of how cut-offs might be applied in a cognitive test, we consider the use of the Montreal Cognitive Assessment (MoCA). In our view, the MoCA is an extremely useful test and we both use it in our clinical practice. It is relatively quick to administer and does not require additional materials other than the test sheet itself. It has been very carefully developed, and is available in many languages and versions. A number of sets of normative data are available. It is widely used. MoCA Cognition states that completion of their Training & Certification Program is necessary to administer, interpret and score test results.

The test sheet itself identifies a normal result as being 26/30 or more. One point may be added to the total score if the individual has ≤12 years of education.

By way of an example of a normative data-set we consider a large community study of the MoCA by Kenny et al (2013) as part of a longitudinal study of older adults. The paper states that people with known dementia, Parkinson's disease or severe cognitive impairment were excluded. The MoCA was administered to 5802 individuals, and the results are presented split by 5-year age bands over age 50, and level of education. Table 2 is from that study. The P values listed in the first column (P95, P90, etc.) are percentiles (not to be confused with the *P*-value in a statistical significance test).

**TABLE 2:** Montreal Cognitive Assessment Scores stratified according to highest educational attainment, based on a sample of 5802 individuals aged 50 and older representative of the community-dwelling population of Ireland without known dementia, Parkinson's disease or severe cognitive impairment

	Age							
Percentile	50	55	60	65	70	75	80	85
Primary or no education								
P95	29	29	29	28	28	28	27	27
P90	28	28	28	28	27	27	26	26
P75	27	26	26	26	26	25	24	23
P50	24	24	24	24	23	22	21	20
P25	22	22	21	21	20	19	18	16
P10	19	19	19	18	17	16	14	12
P05	17	17	17	16	15	13	12	9
Mean ± s.d.	24.0 ± 3.5	23.8 ± 3.6	23.5 ± 3.7	23.1 ± 3.9	22.5 ± 4.1	21.7 ± 4.5	20.7 ± 4.9	19.3 ± 5.5
Secondary education								
P95	29	29	29	29	29	29	28	28
P90	29	29	29	29	28	28	27	27
P75	27	28	27	27	27	26	26	25
P50	26	26	26	26	25	24	23	22
P25	24	24	24	23	23	22	20	18
P10	22	22	21	21	20	19	17	15
P05	20	20	20	19	19	17	15	12
Mean ± s.d.	25.4 ± 2.9	25.4 ± 2.9	25.3 ± 2.9	25.1 ± 3.0	24.6 ± 3.2	23.8 ± 3.6	22.7 ± 4.1	21.1 ± 4.7
Tertiary or higher education								
P95	30	30	30	30	29	29	29	29
P90	30	29	29	29	29	29	28	28
P75	29	29	28	28	28	27	27	27
P50	28	27	27	27	26	26	25	24
P25	26	26	25	25	24	24	23	22
P10	25	24	24	23	22	21	20	19
P05	24	23	22	22	21	20	19	17
Mean ± s.d.	27.3 ± 2.0	27.0 ± 2.1	26 7 ± 2.3	26.3 ± 2 5	25.8 ± 2.7	25.3 ± 2.9	24.7 ± 3.2	23.9 ± 3.5

Source: Kenny et al (2013). Reproduced by kind permission of the American Geriatrics Society.

It can be seen by running the eye along any of the rows that as age increases, scores decrease. This is unlikely to be due to increased prevalence of dementia in older age groups, as those known to have dementia or severe cognitive impairment were excluded. Similarly, by comparing scores in the three education bands for people of similar age it can be seen that scores increase with increasing levels of education. The effects of age and education are very substantial, not only in their effect on median (P50) results within any one group, but even more strikingly when the upper and lower ends (5th and 95th centiles) of the distributions are considered. The effect of education is substantially larger than the 1 point allowance in the published MoCA. The suggested fixed cut-off of 26/30 does not allow any adjustment for age, whereas the normative data suggest that the effects of age between 50 and 85+ account on average for at least a 4 point change and could be as much as 8 points.

Other normative data-sets for the MoCA exist. Another (Rosetti 2011) is a US study of 2653 ethnically diverse individuals, the results also split by age and educational level. The published values are generally slightly lower in the comparable groups than in the Kenny study, but this may be in part because those with known dementia or cognitive impairment were not excluded. As in the Kenny study, the effects of age and educational level on MoCA score are clear.

Wong et al (2015) compared single versus age- and education-corrected cut-off scores using the MoCA in patients after stroke or transient ischaemic attack. They found high levels (>50%) of misclassification when comparing the use of a single fixed cut-off with using age- and education-corrected norms. In other words, using a single fixed cut-off score often identified patients as having normal/abnormal cognitive function differently from using age- and education-adjusted values.

Interestingly, Yeung et al (2020) compared the ability of single cut-off scores (21/22 on the Hong Kong version of the MoCA) versus norm-derived age- and education-adjusted scores in classifying cognitive impairment as normal (cut-off at the 16th centile), mild cognitive impairment (cut-off at 7th centile) or dementia (cut-off at 2nd centile) in a Chinese older adult population. They found that the single cut-off score method had much higher sensitivity than using the 2nd centile norm in classifying people as ‘dementia’ versus ‘normal’ (92.2% *v.* 35.9%), and only slightly lower specificity (92.3% *v.* 98.5%). Their conclusion was that using a fixed cut-off score was a simple and effective method of screening using the MoCA. Although specificity was lower using a fixed cut-off than using age- and education-corrected values, most of the misclassifications were false positives, which meant that individuals could be referred for more careful investigation (as would be expected in a screening process) rather than missed altogether. One reason for the high sensitivity using a fixed cut-off score may have been that (following DSM-5) in the age- and education-adjusted calculation, they used a threshold of 2 standard deviations below the mean to diagnose dementia. As we have seen above, assuming a normal distribution, this means that only 2.5% of that group would be classed as test positive, which is perhaps a much more stringent condition than many clinicians might adopt in practice. It is important to note that using a very stringent norm to diagnose dementia (as DSM-5 suggests) can potentially lead to many missed or delayed diagnoses.

Davis et al (2021) have carried out a Cochrane review of the diagnostic accuracy of the MoCA at various cut-offs for dementia subtypes. They identified four studies that applied the threshold of 26 or above (Smith 2007; Lee 2008; Lu 2011; Larner 2012), as recommended on the MoCA test sheet. In each of these studies, although the sensitivity was high (0.94–1.00), the specificity was low (0.50–0.60). A study with a specificity of 0.5 would mean that of those who tested negative, half would have the disorder and half would not – no better than a random guess. However, in a validation study by Nasreddine et al (2005) (the lead author of the group that created the MoCA test in the mid-1990s) using a cut-off of 25/26, the sensitivity of the MoCA in detecting Alzheimer's dementia was 100% and the specificity was 87%, rather better than in the studies reviewed in the Cochrane paper.

## Hazards of cut-offs

Using a cut-off to interpret a cognitive test is very simple and straightforward, but it can be hazardous. A cut-off may not take into account many of the variables that can influence an individual's performance on a test, as discussed above. A problem that we have been concerned with in this article is that fixed cut-off scores provided for cognitive tests are likely to ignore the effects of age and education, both of which powerfully affect a person's results. Finally, it is important to remember that any cognitive test result relates to only one part of the information that a doctor needs to consider in making a diagnosis. In diagnosing dementia it is essential to consider not only cognitive test results, but also the history and presentation of the patient, the history from an informant, the medical history, blood test results and neuroimaging.

## Conclusions

When choosing a test, we need to think about what it is being used for and whether the one we choose has been adequately validated in a group of people similar to the ones we are testing. We need to be careful about factors that may influence results. In cognitive testing, age and education are very significant factors. Tests that appear to be better or more detailed may lack good normative data and so be more difficult to interpret reliably, although they may still be helpful in tracking changes in a given person over time.

## Data Availability

Data availability is not applicable to this article as no new data were created or analysed in this study.

## MCQs

Select the single best option for each question stem

1. The sensitivity of a test:
  - is a measure of how likely it is that a test result predicts a correct diagnosis
  - is the same as positive predictive value
  - is a measure of how good the test is at identifying all those who have the disorder
  - is the reciprocal of the specificity
  - is the same as the false-negative rate.
2. The specificity of a test:
  - is a measure of how good test is at identifying all those who have the disorder
  - is a measure of how likely it is that if a person tests positive they have the disorder
  - is the proportion of all those who do not have the disease who are test negative
  - is the same as positive predictive value
  - is the same as the false-positive rate.
3. A good cognitive test:
  - has high sensitivity and high specificity
  - has a low area under its receiver operating characteristic (ROC) curve
  - can safely be relied on to make a diagnosis of dementia
  - will not be affected if the patient has difficulty hearing
  - does not need validation.
4. In a normal distribution:
  - approximately 60% of results lie within 2 s.d. of the mean
  - approximately 2.5% of results lie more than 2 s.d. below the mean
  - two-tailed tests give the same result as one-tailed tests
  - the mean and standard error of the mean are the same
  - error bars always show standard deviation.
5. In interpreting a cognitive test result:
  - the age of the person is not relevant
  - the educational level of the person is not relevant
  - it is helpful to compare the result with normative data
  - the test conditions do not matter
  - most people will score the same on consecutive test occasions close together.

MCQ answers 1 c  2 c  3 a  4 b  5 c
